# Impact of Proportional Assist Ventilation Versus Pressure Support Ventilation on Adult Liberation From Mechanical Ventilation: A Revised Systematic Review and Meta‐Analysis of RCTs

**DOI:** 10.1155/ccrp/7536067

**Published:** 2026-10-29

**Authors:** Muhammad Hassan Raza, Obaid Ur Rehman, Umad Ali, Aqsa Komel, Pratishruti, Talha Bin Kashif, Ajay Pandey

**Affiliations:** ^1^ Department of Medicine Nishtar Medical University Multan Pakistan duhs.edu.pk; ^2^ Department of Medicine Services Institute of Medical Sciences Lahore Pakistan sims.edu.pk; ^3^ Department of Medicine King Edward Medical University Lahore Pakistan kemu.edu.pk; ^4^ Mayo Clinic Rochester Minnesota USA mayo.edu; ^5^ Department of Medicine MGM Medical College Navi Mumbai India mgmmedicalcollege.org; ^6^ Department of Medicine Kist Medical College Lalitpur Nepal kistmcth.edu.np

**Keywords:** intensive care unit, mortality, pressure assist ventilation, pressure support ventilation, weaning

## Abstract

**Background:**

Pressure assist ventilation (PAV) is considered superior to pressure support ventilation (PSV). We performed an updated systematic review and meta‐analysis to evaluate comparative effectiveness in terms of weaning outcomes.

**Methods:**

We searched PubMed, Embase, and Cochrane for eligible randomized controlled trials (RCTs) published before October 2025 that directly compared clinical outcomes of PAV and PSV in mechanically ventilated adults. R program was used to calculate risk ratio (RR) and mean difference (MD) with 95% confidence intervals (CIs).

**Results:**

After screening the titles, abstracts, and full texts, we included eight RCTs consisting of 1057 patients in our systematic review and meta‐analysis. PAV was associated with a higher rate of weaning success as compared to PSV (RR: 1.09; 95% CI, 1.01–1.18; *p* = 0.03; *I* ^2^ = 23%). PAV was associated with a lesser duration of ventilation (MD: −1.06; 95% CI, −1.82 to −0.29, *p* = 0.006; *I* ^2^ = 0%) and intensive care unit (ICU) stay (MD: −1.49; 95% CI: −2.43 to −0.54, *p* = 0.002; *I* ^2^ = 25%) as compared to PSV. However, no statistically significant benefit was observed for PAV over PSV with regard to rate of reintubation (RR: 0.82; 95%, CI: 0.58–1.16, *p* = 0.21, *I* ^2^ = 0%) and mortality (RR: 0.89; 95% CI: 0.63–1.25 *p* = 0.4*I* ^2^ = 30%).

**Conclusion:**

PAV is superior to PSV for weaning, showing increased success and shorter duration of ventilation and ICU stays, though reintubation and mortality rates were similar.

## 1. Introduction

Each year, an estimated 20 million patients worldwide require invasive mechanical ventilation (MV), and up to one‐third experience prolonged or difficult weaning or die before successful liberation [1–4]. Acute respiratory failure is a frequent cause of intubation and MV in critically ill patients. Once the underlying condition is stabilized, the principal clinical goal becomes liberation from the ventilator—restoring spontaneous breathing and safely removing the endotracheal tube.

Prolonged dependence on MV can lead to serious complications, including ventilator‐associated pneumonia, ventilator‐induced lung injury, and diaphragmatic dysfunction. The process of discontinuing ventilatory support is guided by a spontaneous breathing trial (SBT), which evaluates the patient’s ability to sustain adequate respiration with little or no assistance. Commonly employed SBT techniques include the T‐piece trial, involving a complete transition to unassisted breathing, and pressure support ventilation (PSV), which provides a gradual reduction in ventilatory assistance [5]. PSV is often used to help patients breathe on their own before removing the ventilator. However, because it gives a fixed level of help, it may not adjust well to changes in the patient’s breathing effort, which can cause mismatches and slow the weaning process. Pressure/proportional assist ventilation (PAV), terms used interchangeably, adjusts support in real time to match the patient’s effort, improving comfort and helping patients come off the ventilator more easily. If not properly adjusted, PSV can result in excessive assistance, diaphragm weakness, and patient–ventilator asynchrony, all of which may contribute to prolonged weaning [6–9].

Many studies in the past have tried to answer the question of whether PAV or PSV is better for weaning from invasive MV. Most recent is the meta‐analysis by Ou‐Yang et al. [10]; however, a more robust randomized controlled trial (RCT) by Bosma et al. has been published in 2025 on the same topic which has sample size almost equivalent to sample size of all previous RCTs combined in the past (included in Ou‐Yang et al. [10] to synthesize data). This is the reason this update is going to be significant (Table 1 and Figure 1).

**TABLE 1:** Comparison of the primary and secondary outcomes reported in the previous meta‐analysis and the addition of new RCT, Bosma et al. [11], to synthesize updated data (RR: relative risk; MD: mean difference; AD: absolute difference).

Study		Reintubation rate	Mortality	ICU stay (days)	Assist control mode use	Duration of ventilation (hrs)
Ou‐Yang et al. [10] (previous meta‐analysis)	PAV vs. PSV	RR, 0.49 [0.28, 0.87]	RR, 0.66 [0.42, 1.06]	MD, −1.58 [−2.6, −0.47]	—	MD, −1.68 [−2.77, −0.57]
Bosma et al. [11] (new RCT)	PAV vs. PSV	AD, −1.1 [−8.6, 6.4]	AD, 3.4 [−4.0, 10.7]	AD, 0.8 [−2.9, 4.2]	AD, 9.2 [1.1, 17.4]	AD, 0.6 [0.2, 1.2]
Updated analysis results	PAV vs. PSV	RR, 0.82 [0.581, 16]	RR, 0.89 [0.63, 1.25]	MD, −1.49 [−2.43, −0.54]	RR, 0.72 [0.31, 1.6]	MD, −1.06 [−1.82, 0.29]

**FIGURE 1:**
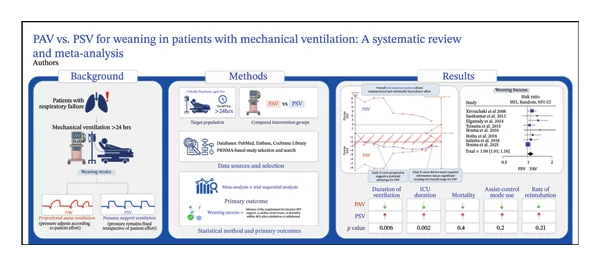
Pooled analysis of randomized controlled trials (RCTs) comparing proportional assist ventilation (PAV) vs. pressure support ventilation (PSV) for weaning success.

## 2. Methods

This meta‐analysis was conducted following the Preferred Reporting Items for Systematic Reviews and Meta‐Analyses (PRISMA) guidelines. The protocol is registered with the International Prospective Register of Systematic Reviews (PROSPERO) under the identifier CR (CRD420251181637). Ethical approval was not necessary for this study since it exclusively utilized publicly available data.

### 2.1. Data Sources and Searches

We searched PubMed, Embase, and the Cochrane Library to identify all relevant trials, by screening titles and reviewing abstracts, with no filters or language restrictions. In order to ensure that no RCTs were missing, conference abstracts and reference lists of included articles were reviewed. We searched for ongoing trials, using Google Scholar and the US government clinical trials database (https://www.ClinicalTrials.gov). Two independent investigators Umad Ali and Aqsa Komel conducted a systematic search for RCTs published up until November 2025, using the terms “Proportional Assisted Ventilation,” “Pressure Support Ventilation,” “Ventilation Weaning,” and “Spontaneous Breathing Trial” (Supporting file, Table S1).

### 2.2. Eligibility Criteria

Inclusion criteria were based on the PICOS framework (Supporting file, Table S2). We selected RCTs that included adults (aged 18 years or older) with respiratory failure from various causes who had received invasive MV for 24 h or more and were undergoing weaning trials (using PAV or PAV + vs. PSV) for liberation from ventilation. Studies published in English and Chinese language were selected. The exclusion criteria were as follows: trials investigating neonatal or pediatric patients (patients aged less than 18 years), trials that extubated patients directly to noninvasive ventilation for weaning purposes, trials with no active comparison group (i.e., placebo or no treatment), and trials that compared the same ventilation mode but had different parameters.

### 2.3. Data Extraction and Bias Assessment

Two reviewers (Umad Ali and Aqsa Komel) independently extracted the data from all included articles. Data extraction was performed to capture information on study‐related, participant‐related, and treatment‐related characteristics. Authors of studies eligible for inclusion in our review were contacted if original data were missing. The quality of the RCTs was appraised by Muhammad Hassan Raza and Obaid Ur Rehman using the Cochrane Risk of Bias tool. Assessments of the risks of selection bias (random sequence generation and allocation concealment), performance bias (blinding of participants and personnel), detection bias (blinding of outcome assessment), attrition bias (incomplete outcome data), reporting bias (selective reporting), and other bias were appraised as high, unclear, and low risk (Supporting file Figure S1). Any disagreement was resolved via group discussions.

### 2.4. Outcome Measures

We extracted data on six outcomes. The primary outcome was weaning success, defined as the absence of the requirement for invasive MV support, a cardiac arrest event, or mortality within 48 h after extubation. The secondary outcomes were as follows: the proportion of patients requiring reintubation (defined as the patient requiring reintubation within 48 h after extubation), overall mortality, the proportion of patients undergoing assist‐control mode use (defined as the patient requiring assist‐control mode use after extubation failure), intensive care unit (ICU) length of stay (the time from admission to live ICU discharge), and ventilation duration (the time from intubation to extubation).

### 2.5. Data Synthesis and Analysis

We analyzed the data as recommended in the Cochrane Handbook for Systematic Reviews of Interventions. We analyzed dichotomous variables, using the Mantel–Haenszel method and DerSimonian–Laird estimator, and calculated risk ratios (RRs) with 95% confidence intervals (CIs). To measure continuous outcomes, we employed the inverse variance method and DerSimonian–Laird estimator and calculated the mean difference (MD) with 95% CIs. Administration of fixed effect or random effects was interpreted with statistical heterogeneity by authors. If an estimate of the between‐study variance, known as tau‐squared, was low (or zero), then we would choose a fixed‐effect model; otherwise, we would choose random effects. Statistical heterogeneity was evaluated using Cochran’s *Q* test, with a significance threshold set at *p* < 0.10, and quantified using I^2^ statistic, where *I* ^2^ values of 25%, 50%, and 75% were interpreted as low, moderate, and high, respectively. Potential publication bias and small study effects were assessed through the generation of Doi plots and calculation of the Luis Furuya‐Kanamori (LFK) index, which offers a quantitative measure of asymmetry. An LFK index between −1 and +1 is considered indicative of symmetry (no publication bias), values between ±1 and ± 2 suggest minor asymmetry, and values beyond ±2 denote major asymmetry. A two‐tailed *p* < 0.05 was considered statistically significant for all tests unless otherwise specified. We conducted subgroup analysis by the types of different proportional modes, PAV and PAV+, and examined the differences in outcomes between these subgroups by testing for interactions. A leave‐one‐out sensitivity analysis was performed to assess the robustness of the pooled estimate. This involved sequentially excluding each study and recalculating the overall prevalence estimate to determine the influence of each study on the meta‐analytic summary measure. All statistical analyses were performed using the “metafor” and “meta” packages of R software Version 3.6.1. A *p* value of < 0.05 was taken to indicate statistical significance.

### 2.6. Quality Assessment

The Grading of Recommendations Assessment, Development, and Evaluation (GRADE) methodology was used to assess the certainty of evidence from the included studies (GRADEpro, Version 20; McMaster University, 2014).

### 2.7. Trial Sequential Analysis

Sparse data and repetitive testing of accumulating data in meta‐analyses can produce an increased risk of both Type 1 and Type 2 errors. Therefore, TSA was conducted to challenge the meta‐analysis, in case the data were too sparse to confirm the conclusions, and avoid early overestimates by combining the estimated required information size with an adjusted threshold. We constructed TSA boundaries, according to the O’Brien–Fleming alpha‐spending function, to assess whether the *p* value was statistically significant enough to show the anticipated effect or whether the analysis should be terminated early. A 5% (*α* = 0.05; two‐sided) total risk of Type 1 error and 80% statistical power were set. We assumed a relative risk reduction of 10% for the primary outcome of weaning success. The event rate in the control group was calculated from the mean of the event proportions in the low‐risk‐of‐bias trials. We provide the TSA‐adjusted 95% CIs. Fixed‐effect TSA was performed using trial sequential analysis software (Version 0.9.5.10 Beta; Copenhagen Trial Unit, Copenhagen, Denmark).

## 3. Results

### 3.1. Included Studies

The PRISMA statement flowchart (Figure 2) outlines the literature screening process, study selection, and exclusion criteria. The initial search yielded 634 articles, from which 75 full‐text articles were retrieved for assessment. Ultimately, 8 studies met the eligibility criteria and were included in our meta‐analysis.

**FIGURE 2:**
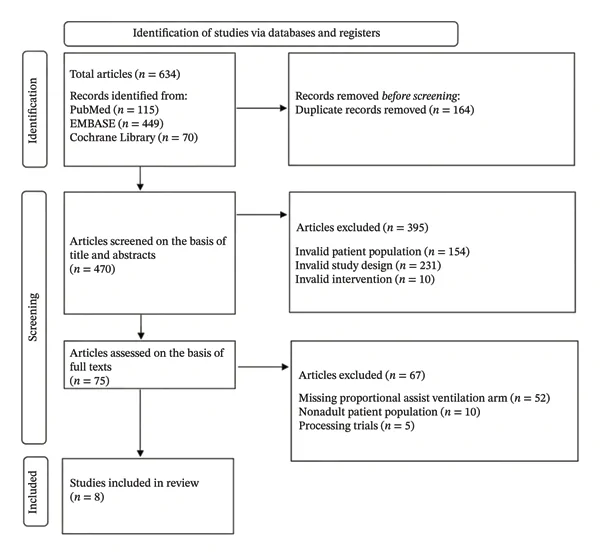
PRISMA flowchart.

### 3.2. Study Characteristics

Studies spanned from 2008 to 2025 and were conducted in multiple centers all over the world. Sample sizes ranged from 23 to 722 patients. The pooled population in the PAV group was 538 while 519 for PSV group. The mean age for patients ranged from 44.5 to 64.8 years. Male participants made up 40%–80% of the samples.7 studies used PAV + while 1 study used PAV as intervention. 4 studies used APACHE II as physiology score, and 1 study used APACHE III while 3 studies did not report physiology score. Detailed characteristics of each study can be found in Table 2.

**TABLE 2:** Baseline characteristics of patients.

Authors	Year	Design	Country	Sample size	Mean age (years)	Male (%)	Mean MV duration (days)	Physiology score	Principal population
Xirouchaki et al.	2008	RCT	Greece	208	60.9	66.3	4.0	APACHE II: 15.5	Mixed ICU

Sasikumar et al.	2013	RCT	India	23	48.6	69.6	NR	APACHE II: 20.7	Mixed critical illness

Elganady et al.	2014	RCT	Egypt	60	59.7	81.7	NR	NR	AECOPD

Teixeira et al.	2015	RCT	Brazil	160	44.5	65.6	6.6	APACHE II: 22.7	Mixed ICU

Bosma et al.	2016	RCT	Canada	50	64.8	50.0	5.8	APACHE II: 26.5	Mixed ICU

Botha et al.	2018	RCT	Australia	50	63.2	59.2	3.4	APACHE III: 76.7; SAPS: 45.5	Mixed ICU

Salama et al.	2018	RCT	Egypt	150	NR	NR	NR	NR	Mixed ICU

Bosma et al.	2025	RCT	Argentina Canada France Greece Italy Saudi Arabia Spain	722	61.2	63.2	4.9	NR	Mixed ICU

Abbreviation: NR, not reported.

### 3.3. Quality Assessment

The risk of bias in the included studies in five domains, i.e., selection bias, performance bias, detection bias, attrition bias, and reporting bias, was assessed using RoB 2.0 software by the Cochrane Collaboration. Overall risk of bias was low in 1 study only, and 4 studies had unclear risk while 3 studies were found to be high risk. The traffic light plot and summary plot are depicted in Figure 3 and supporting file Figure S1.

**FIGURE 3:**
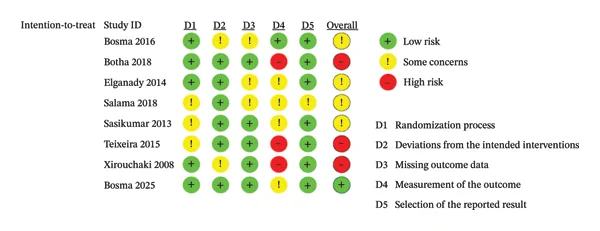
Traffic light plot showing risk of bias across five domains and its overall effect on the study.

## 4. Outcomes

### 4.1. Weaning Success

Our pooled analysis included 8 studies, and it was found that the rate of weaning success was significantly greater in the PAV group compared to the PSV group (RR: 1.09; 95% CI: 1.01–1.18, *p* = 0.03) (Figure 4). The heterogeneity was found to be low (*I* ^2^ = 23%).

**FIGURE 4:**
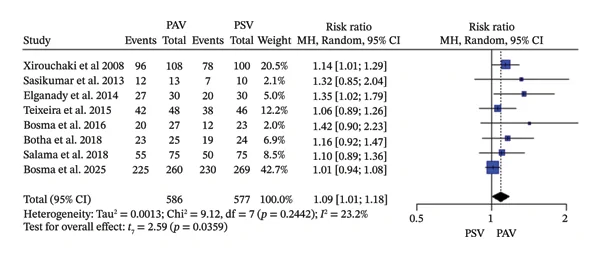
Forest plot comparing weaning success between proportional assist ventilation (PAV) and pressure support ventilation (PSV). The pooled analysis of eight studies demonstrated a significantly higher rate of weaning success in the PAV group compared with the PSV group (risk ratio [RR] = 1.09; 95% confidence interval [CI]: 1.01–1.18; *p* = 0.03). Between‐study heterogeneity was low (*I* ^2^ = 23%).

### 4.2. Duration of Ventilation

Six studies reported this outcome, and our pooled analysis showed that the duration of ventilation was significantly less in the PAV group compared to the PSV group (MD: −1.06; 95% CI: −1.82 to −0.29, *p* = 0.006) (Figure 5). The heterogeneity was found to be very low (*I* ^2^ = 0%).

**FIGURE 5:**
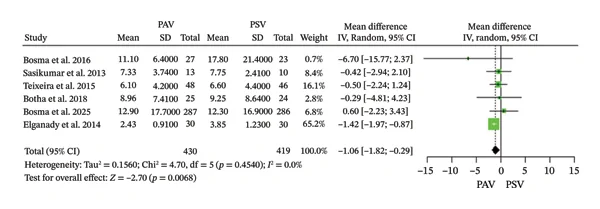
Forest plot comparing duration of mechanical ventilation between proportional assist ventilation (PAV) and pressure support ventilation (PSV). Pooled analysis of six studies showed that the duration of ventilation was significantly shorter in the PAV group compared with the PSV group (mean difference [MD] = −1.06; 95% confidence interval [CI]: −1.82 to −0.29; *p* = 0.006). No significant heterogeneity was observed among studies (*I* ^2^ = 0%).

### 4.3. ICU Duration

Our pooled analysis showed that ICU duration was significantly less in the PAV group compared to the PSV group (MD: −1.49; 95% CI: −2.43 to −0.54, *p* = 0.002) (Figure 6). The heterogeneity was found to be low (*I* ^2^ = 25%).

**FIGURE 6:**
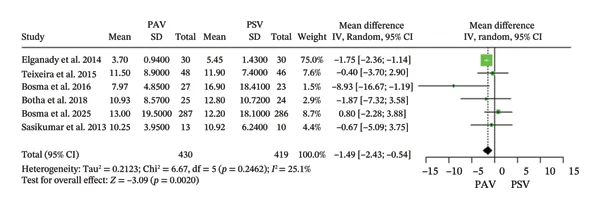
Forest plot comparing intensive care unit (ICU) length of stay between proportional assist ventilation (PAV) and pressure support ventilation (PSV). The pooled analysis demonstrated a significantly shorter ICU duration in the PAV group compared with the PSV group (mean difference [MD] = −1.49; 95% confidence interval [CI]: −2.43 to −0.54; *p* = 0.002). Between‐study heterogeneity was low (*I* ^2^ = 25%).

### 4.4. Mortality

Mortality was less in patients undergoing PAV as compared to PSV (RR: 0.89; 95% CI: 0.63–1.25 *p* = 0.4*I* ^2^ = 30%) (Figure 7). However, the results were not statistically significant.

**FIGURE 7:**
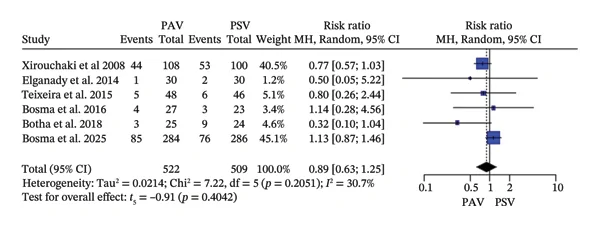
Forest plot comparing mortality between patients managed with proportional assist ventilation (PAV) and pressure support ventilation (PSV). The pooled analysis showed a lower mortality rate in the PAV group compared with the PSV group; however, this difference was not statistically significant (risk ratio [RR] = 0.89; 95% confidence interval [CI]: 0.63–1.25; *p* = 0.40). Between‐study heterogeneity was low to moderate (*I* ^2^ = 30%).

### 4.5. Assist‐Control Mode Use

It was seen that assist‐control mode was used more in the PSV group as compared to the PAV group (RR: 0.72; 95% CI: 0.31–1.67, *p* = 0.2, *I* ^2^ = 78.7%) (Figure 8). However, the results were not statistically significant.

**FIGURE 8:**
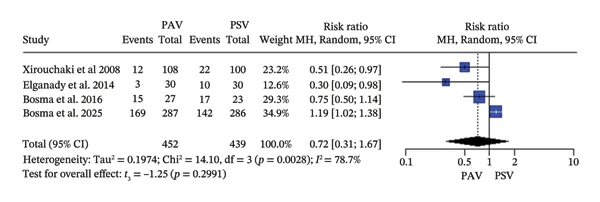
Forest plot comparing the use of assist‐control ventilation between the proportional assist ventilation (PAV) and pressure support ventilation (PSV) groups. Assist‐control mode was used more frequently in the PSV group compared with the PAV group; however, the difference was not statistically significant (risk ratio [RR] = 0.72; 95% confidence interval [CI]: 0.31–1.67; *p* = 0.20). Substantial heterogeneity was observed among the included studies (*I* ^2^ = 78.7%).

### 4.6. Rate of Reintubation

Our pooled analysis showed that the rate of reintubation was less in the PAV group compared to the PSV group (RR: 0.82; 95%, CI: 0.58–1.16, *p* = 0.21, *I* ^2^ = 0%) (Figure 9). However, the results were not statistically insignificant.

**FIGURE 9:**
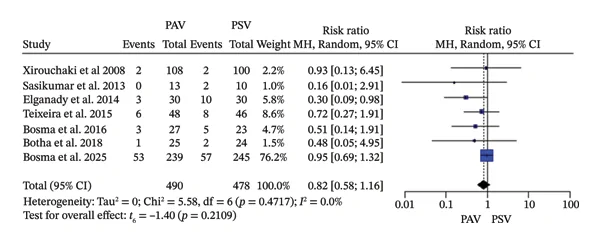
Forest plot comparing reintubation rates between proportional assist ventilation (PAV) and pressure support ventilation (PSV). The pooled analysis showed a lower rate of reintubation in the PAV group compared with the PSV group; however, this difference was not statistically significant (risk ratio [RR] = 0.82; 95% confidence interval [CI]: 0.58–1.16; *p* = 0.21). No heterogeneity was observed among studies (*I* ^2^ = 0%).

### 4.7. Publication Bias

Publication bias across all 6 outcomes was assessed using Doi plots (Figure 10) along with LFK index. The LFK index quantifies this deviation, with values between −1 and +1 indicating no asymmetry and low bias, values between ±1 and ±2 suggesting minor asymmetry and moderate bias, and values exceeding ±2 indicating major asymmetry and high publication bias. Weaning success (LFK index: +5.19), assist‐control mode (LFK index: −6.57), and rate of reintubation (LFK index: −5.63) showed major asymmetry in Doi plots suggesting substantial publication bias. Duration of ventilation (LFK index: +1.38) and mortality (LFK index: ±1.93) showed minor asymmetry in graph suggesting moderate bias while ICU duration (LFK index: −0.47) had a symmetric graph indicating low publication bias. Overall Doi–LFK evaluation suggested that several outcomes were affected by small study effects and selective publication bias, which may influence the certainty of the pooled estimates (supporting file Figure S4A–S4E). However, the total number of included studies was limited to seven, which further restricts the power of publication bias assessment and increases the likelihood of small‐study effects.

**FIGURE 10:**
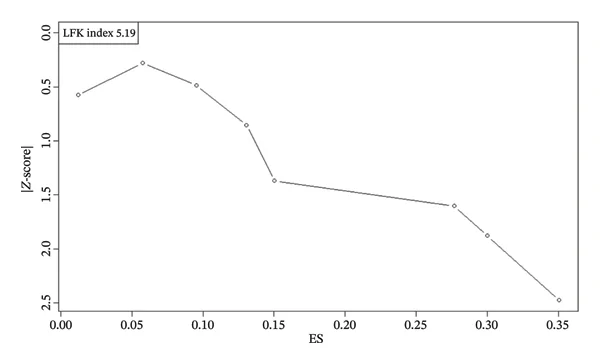
Doi plot assessing publication bias for the outcome of weaning success. The plot demonstrates marked asymmetry, with an LFK index of 5.19, indicating major asymmetry and a high likelihood of publication bias or small‐study effects among the included studies. The *x*‐axis represents the effect size (ES), and the *y*‐axis represents the standardized *Z*‐score (supporting file Figure S4A–S4E).

### 4.8. Sensitivity Analysis and Subgroup Analysis

Sensitivity analysis was performed using the leave‐one‐out method for 4 outcomes: weaning success (Figure 11), mortality, ICU duration, and use of assist‐control mode. The results did not vary significantly upon excluding studies, and no outlier study was found in all these outcomes, thus proving the robustness of our findings.

**FIGURE 11:**
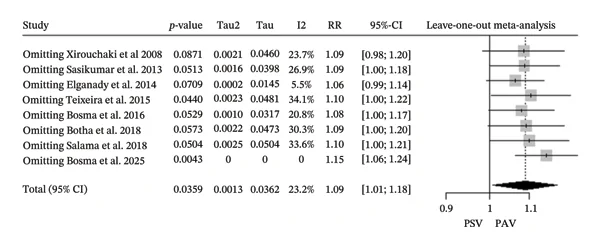
Leave‐one‐out sensitivity analysis for the outcome of weaning success comparing proportional assist ventilation (PAV) with pressure support ventilation (PSV). Each row represents the pooled risk ratio (RR) and heterogeneity estimates after sequential omission of an individual study. The overall effect remained consistent across omissions, with pooled estimates favoring PAV, indicating that no single study disproportionately influenced the results. The final pooled analysis showed a significantly higher weaning success rate with PAV (RR = 1.09; 95% CI: 1.01–1.18) with low heterogeneity (*I* ^2^ = 23.2%), demonstrating robustness of the findings.

A subgroup analysis was performed by dividing the studies into two groups based on types of PAV. In the subgroup of patients undergoing PAV+, we found a greater chance of weaning success. A similar trend was seen in the subgroup of patients undergoing PAV. However, the subgroup analysis of mortality, ICU duration, and proportion of patients requiring reintubation was statistically insignificant for both PAV and PAV+ (Figure 12 and supporting file figure S3A–S3C).

**FIGURE 12:**
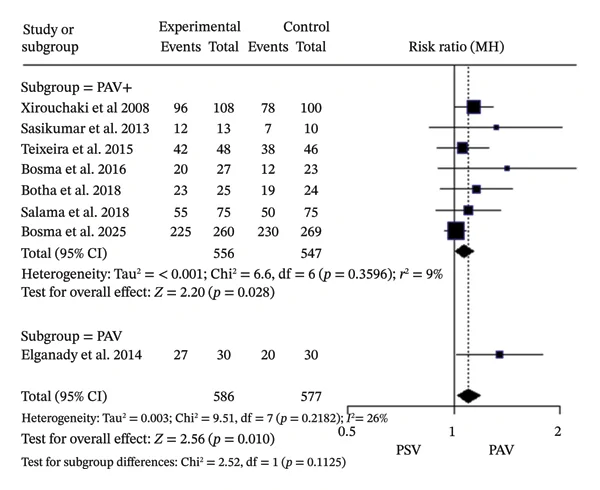
Subgroup analysis forest plot comparing weaning success between proportional assist ventilation (PAV) and pressure support ventilation (PSV). Studies were stratified into PAV+ and PAV subgroups. In the PAV + subgroup, pooled analysis demonstrated a significantly higher weaning success rate with PAV compared to PSV, with low heterogeneity (*I* ^2^ = 9%). The PAV subgroup also favored PAV, with moderate heterogeneity (*I* ^2^ = 26%). The overall effects across all studies showed a significant benefit of PAV over PSV. No statistically significant difference was observed between subgroups (test for subgroup differences: *χ* ^2^ = 2.52, *p* = 0.11), indicating consistency of the effect across ventilation modes (supporting file Figure S2A–S2C).

### 4.9. Trial Sequential Analysis

TSA of the primary outcome, weaning success, is shown in Figure 13. In this TSA, the required information size was calculated allowing for an overall Type 1 error of 5% and a Type 2 error of 20% and assuming a control event proportion of 0.78, a relative risk reduction of −8.52% for the effect size, and a model variance‐based estimate of diversity. The estimates for the control event proportion and diversity were taken from the included trials, as described in the “Methods” section. TSA of weaning success showed that the effect size of 1163 did not exceed the required information size of 2110 patients. The TSA‐adjusted CI was 1.09 (95% CI 1.03, 1.15). When the accumulated data were less than the required information size, the cumulative z‐curve needed to cross the monitoring boundaries to declare either significance or futility. The cumulative z‐curve crossed the conventional boundary and the trial sequential monitoring boundary for benefit in favor of PAV, suggesting conclusive, robust results. Therefore, TSA confirmed our meta‐analysis providing convincing statistical evidence (supporting file S5A–S5E). The TSA results of all secondary outcomes demonstrated inconclusive results with sparse data except ventilation duration, which are described in supporting file 1.

**FIGURE 13:**
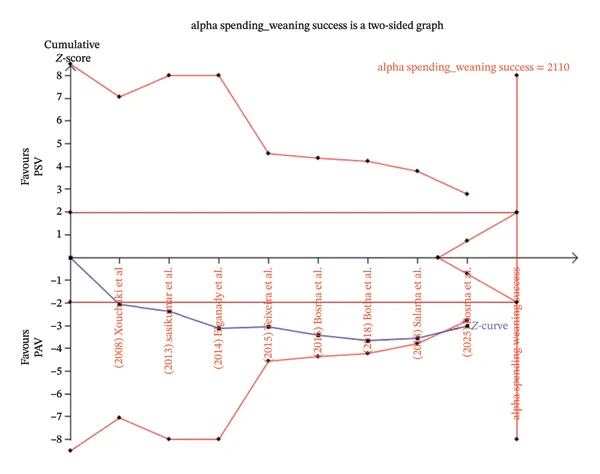
Trial sequential analysis (TSA) of the primary outcome, weaning success. TSA was performed with a Type I error of 5%, Type II error of 20%, control event proportion of 0.78, relative risk reduction of −8.52%, and diversity estimated from included trials. The accumulated sample size (*n* = 1163) did not reach the required information size (*n* = 2110). However, the cumulative *Z*‐curve crossed both the conventional and trial sequential monitoring boundaries for benefit, favoring PAV, with a TSA‐adjusted RR of 1.09 (95% CI 1.03–1.15), indicating robust and conclusive evidence. Secondary outcomes showed inconclusive TSA results due to sparse data (see Additional file 1).

## 5. Discussion

Proportional assist ventilation (PAV) offers only modest or mixed benefits compared to PSV in facilitating weaning from MV in adult patients. The most recent and largest multicenter randomized trial (PROMIZING) found no statistically significant difference in time to successful liberation from MV between PAV and PSV when both modes were protocolized and carefully titrated, with median weaning times of 7.3 days for PAV and 6.8 days for PSV (*p* = 0.58) [11]. Secondary outcomes—including ventilator‐free days, reintubation rates, tracheostomy rates, and 90‐day mortality—were also similar between groups.

Earlier meta‐analyses and smaller trials suggested that PAV may increase weaning success rates and reduce duration of MV and ICU length of stay by approximately 1‐2 days compared to PSV [10, 12, 13]. However, these benefits are less pronounced in recent high‐quality studies, and publication bias may have inflated prior estimates [10–12]. Heterogeneity across studies is low (*I* ^2^ = 23%), supporting the reliability of pooled results, but the magnitude of benefit should be interpreted cautiously.

Physiologically, PAV reduces patient–ventilator asynchrony and dyspnea more effectively than PSV, but these improvements do not consistently translate into faster weaning in rigorously controlled protocols [13, 14]. PSV remains the most widely used partial support mode, but its fixed pressure delivery can result in mismatches with patient effort, potentially prolonging ventilation in some cases [14, 15]. Unlike PSV (which delivers a fixed preset pressure), PAV delivers assistance proportional to the patient’s own inspiratory effort, using closed‐loop measurements of respiratory‐system compliance and resistance, which theoretically affords several benefits [14].

Based on our analysis, proportional assist ventilation (PAV) offers a statistically significant but modest reduction in both MV duration and ICU length of stay compared to PSV. The magnitude of benefit is relatively small, typically amounting to a reduction of approximately 1‐2 days in ICU stay and 40 h in MV duration, as demonstrated in pooled analyses with minimal heterogeneity However, large multicenter trials have shown that these differences are not always clinically significant, with absolute differences in time to liberation from MV and ICU discharge often less than one day and not reaching statistical significance in rigorously controlled settings [11].

The clinical impact of PAV is therefore limited, and while improved patient–ventilator synchrony and reduced asynchrony are consistent physiological advantages, these do not consistently translate into meaningful improvements in patient‐centered outcomes such as mortality, reintubation rates, or long‐term functional status [11, 15, 16]. The modest reduction in duration should be interpreted in the context of overall weaning protocols and patient selection, as the choice of ventilatory mode may be less important than systematic approaches to weaning [11].

Current evidence from recent meta‐analyses and RCTs indicates that proportional assist ventilation (PAV or PAV) does not significantly reduce ICU mortality or hospital mortality compared to PSV or other advanced modes. The PROMIZING trial (Bosma et al.) found no significant difference in 90‐day mortality between PAV and PSV (29.6% vs. 26.6%, *p* = 0.58), and similar findings were reported in meta‐analyses by Ou‐Yang et al. and Tirupakuzhi Vijayaraghavan et al., which showed no statistically significant effect of PAV on hospital mortality or reintubation rates compared to PSV [10, 11, 15]. Network meta‐analysis by Wu et al. further supports that PAV does not confer a mortality benefit over PSV or other modes, while NAVA may reduce ICU mortality compared to both PSV and PAV [17]. Kampolis et al. also found no significant difference in ICU or hospital mortality between PAV and PSV, though neurally adjusted ventilatory assist (NAVA) was associated with improved survival [12].

Regarding escalation to assist‐control ventilation, the PROMIZING trial noted that switches to assist‐control mode occurred more frequently with PAV than with PSV, potentially reflecting clinician unfamiliarity with PAV in some centers [11]. In contrast, Xirouchaki et al. found a lower failure rate (need for escalation) with PAV compared to PSV in a single‐center study, highlighting the impact of institutional protocols and experience [18].

Interpretation of these outcomes is limited by small sample sizes in many studies, potential publication bias favoring positive results, and substantial variability in weaning protocols and clinician expertise across institutions. These factors may obscure true differences in mortality and escalation rates, and the modest reductions in ventilation and ICU duration observed with PAV are not accompanied by clinically meaningful improvements in survival or major adverse events [10, 11, 17, 18].

Although earlier meta‐analyses have reported small reductions in ventilation duration and ICU length of stay with PAV, the absolute differences are minimal and unlikely to be clinically meaningful for most patients [17]. Moreover, concerns about publication bias and variability in weaning approaches across studies limit the applicability of these results [11, 17]. Importantly, the stringent protocol and standardized weaning procedures used in the Bosma et al. trial helped reduce confounding and suggest that consistent weaning practices may play a more decisive role than the specific ventilatory mode itself [11].

Overall, current evidence does not indicate that PAV provides a substantial clinical advantage over PSV in terms of reintubation rates, total ventilator days, or ICU stay. Although PAV offers theoretical physiological benefits, such as better patient–ventilator synchrony, these improvements do not reliably lead to better patient‐centered outcomes. Future studies should aim to identify patient subgroups who might benefit more from personalized ventilatory strategies [11, 15, 17].

### 5.1. Strengths and Limitations

This meta‐analysis offers several important strengths. Foremost, it integrates evidence from multiple RCTs, thereby enhancing the reliability and generalizability of its conclusions. Additionally, key outcomes—such as duration of MV and ICU length of stay—demonstrated low heterogeneity, suggesting consistent effects across studies. The application of sensitivity analyses and comprehensive risk‐of‐bias assessments further strengthens the robustness of the findings.

Nonetheless, several limitations warrant consideration. Evidence of publication bias was identified for multiple outcomes, raising the possibility that effect sizes may be overestimated. Sample sizes were limited for critical endpoints such as mortality and reintubation, reducing statistical power to detect meaningful differences. Moreover, variations in ventilatory settings, protocol designs, and definitions of weaning success across studies may have contributed to residual heterogeneity and constrained the comparability of results. These factors should be acknowledged when interpreting the findings and applying them to broader clinical settings.

Future research should prioritize large, multicenter randomized trials to more definitively evaluate the impact of PAV on mortality, extubation failure, and long‐term clinical outcomes.

The adoption of standardized weaning protocols and objective measures of patient–ventilator synchrony would improve comparability and strengthen mechanistic understanding. Additionally, prespecified subgroup analyses are needed to identify patient populations—such as individuals with COPD or those who are difficult to wean—who may derive the greatest benefit, thereby informing more tailored and effective clinical practice.

## Funding

No funding was received for this manuscript.

## Conflicts of Interest

The authors declare no conflicts of interest.

## Supporting Information

Additional supporting information can be found online in the Supporting Information section.

## Supporting information


**Supporting Information** The supporting files provide additional methodological details and support analyses for this review. Table S1 summarizes the detailed search strategy used across all databases. Table S2 summarizes the PICOS framework. Figures S1 to S5 include supporting visual materials. Figure S1. Assessment of risk of bias. Figure S2. Subgroup analysis of outcomes. Figure S3. Sensitivity analysis of outcomes. Figure S4. Doi plots and Eager’s test. Figure S5. Trial sequential analysis of secondary outcomes.

## Data Availability

The data that support the findings of this study are available from the corresponding author upon reasonable request.
