# Supplementary material for: Impact of Proportional Assist Ventilation Versus Pressure Support Ventilation on Adult Liberation From Mechanical Ventilation: A Revised Systematic Review and Meta‐Analysis of RCTs
Source: Crit Care Res Pract. 2026 Oct 29;2026:7536067. doi: 10.1155/ccrp/7536067 (PMC13717589; doi:10.1155/ccrp/7536067)
Supplement: Supplementary file 1 [file CCRP-2026-7536067-s001.zip › PICOS table supplementary file.docx]

# PICOS Framework

| Component | Description |
| --- | --- |
| Population (P) | Adult patients (≥18 years) with respiratory failure from any cause who received invasive mechanical ventilation for at least 24 hours and were undergoing weaning from mechanical ventilation. |
| Intervention (I) | Proportional assist ventilation (PAV) or proportional assist ventilation plus (PAV+). |
| Comparator (C) | Pressure support ventilation (PSV). |
| Outcomes (O) | The primary outcome was weaning success. Secondary outcomes included duration of mechanical ventilation, intensive care unit (ICU) length of stay, reintubation rate, mortality, and requirement for assist-control ventilation. |
| Study Design (S) | Randomized controlled trials (RCTs). |
