# Supplementary material for: Impact of Proportional Assist Ventilation Versus Pressure Support Ventilation on Adult Liberation From Mechanical Ventilation: A Revised Systematic Review and Meta‐Analysis of RCTs
Source: Crit Care Res Pract. 2026 Oct 29;2026:7536067. doi: 10.1155/ccrp/7536067 (PMC13717589; doi:10.1155/ccrp/7536067)
Supplement: Supplementary file 1 [file CCRP-2026-7536067-s001.zip › PAV vs PSV supplementry file.docx]

**Supplementary Information - Additional file 1**

**Impact of Proportional Assist Ventilation Versus Pressure Support Ventilation on Adult Liberation from Mechanical Ventilation: A Revised Systematic Review and Meta‑Analysis of RCTs**

Muhammad Hassan Raza, Obaid Ur Rehman, Umad Ali, Aqsa Komel, FNU Pratishruti, Talha Bin Kashif

**Contents**

Table S1. Search strategy

Figure S1. Assessment of risk of bias

Figure S2. Subgroup analysis of outcomes

Figure S3. Sensitivity analysis of outcomes

Figure S4. Doi plots and Eager’s test.

Figure S5. Trial sequential analysis of secondary outcomes

**S1. Search Strategy**

Supplementary Table 1: Search strategy utilized in various databases included in the systematic review (Conception of databases till November 2025)

| **Database** | **Search Strategy** |
| --- | --- |
| MEDLINE via PubMed  (115 Results) | (((((("weaning"[All Fields] OR "spontaneous breathing test"[All Fields]) OR "spontaneous breathing trial"[All  Fields]) OR "sbt"[All Fields]) OR "spontaneous breathing"[All Fields]) OR (("ventilator"[All Fields] OR  "mechanical ventilation"[All Fields]) OR "ventilation"[All Fields])) AND (("pressure support"[All Fields] OR  "pressure support mode"[All Fields]) OR "pressure support ventilation"[All Fields])) AND ((((("proportional  assist ventilation"[All Fields] OR "proportional assist ventilator"[All Fields]) OR "proportional assist"[All Fields])  OR "proportional assisted ventilation"[All Fields]) OR "PAV"[All Fields]) OR "PAV+"[All Fields]) |
| Cochrane Library  (70 Results) | Weaning AND Pressure Support Ventilation AND Proportional Assist Ventilation |
| EMBASE  (449 Results) | weaning AND spontaneous breathing test/trial AND spontaneous breathing trial OR spontaneous breathing AND mechanical ventilation AND pressure support mode or pressure support ventilation AND proportional assist ventilation OR proportional assist ventilator OR proportional assist OR proportional assisted ventilation OR pav OR pav+ |

**Figure S1: Assessment of risk of bias.**

**Figure S2: Subgroup analysis of outcomes:**

**Figure S2A ICU duration:**

This illustration represents the risk of biased judgments, expressed as percentages within an intention-to-treat framework, spanning important methodological areas. While most studies show a low risk of selecting the reported result and deviating from targeted interventions, numerous domains—including the randomization method, missing outcome data, and outcome measurement—reveal significant proportions of some concerns and risk. Overall, the chart demonstrates diversity in methodological quality, with outcome measurement and overall bias being the most readily apparent indicators of potential bias.

**Figure S2B Mortality:**

This forest plot depicts the pooled mean differences between experimental and control groups across studies, divided into subgroups (PAV and PAV+). Individual research effects with 95% confidence intervals are displayed with subgroup and overall pooled estimates based on a random-effects model. The analysis shows low to moderate heterogeneity between subgroups, with no statistically significant overall effect or differences between subgroups, showing that PAV and PSV techniques produce comparable results.

**Figure S2C Patients requiring intubation:**

The forest plot - comparing proportional assist ventilation (PAV/PAV+) to pressure support ventilation (PSV), shows individual study risk ratios with associated event counts, sample sizes, and 95% confidence intervals. The majority of studies provide effect estimates that cross the line of no effect, showing that neither ventilation mode provides a consistent advantage. The pooled analysis found no statistically significant difference between PAV (or PAV+) and PSV, with low to high heterogeneity and no significant subgroup differences. Overall, the findings indicate that PAV-based modes and PSV had equivalent results throughout the research examined.

**Figure S3: Sensitivity Analysis:**

This forest plot compares proportional assist ventilation (PAV/PAV+) and pressure support ventilation (PSV), depicts risk ratios reported with 95% confidence intervals across individual studies and designated subgroups. The majority of study estimates have tight confidence ranges and little statistical heterogeneity (I² = 0%), indicating a lack of effect. The combined results for the PAV+ subgroup and the overall study show no statistically significant difference between PAV-based modes and PSV. Overall, our findings suggest that ventilation techniques produce equivalent risk outcomes, with consistent effects across studies and no significant subgroup differences.

**Figure S3A: ICU duration**

The leave-one-out sensitivity analysis reveals that the pooled effect estimate continuously favors PAV over PSV, with negative mean differences reported after sequentially excluding individual studies. The direction and amount of the effect stay rather constant throughout iterations, and no single study has a significant impact on the overall conclusions. The level of heterogeneity remains low to moderate, indicating that the meta-analysis' conclusions are strong and stable.

**Figure S3B: Mortality**

**The sequential removal of individual studies did not significantly impact the pooled risk ratio or its statistical importance. The overall effect consistently remained non-significant in all iterations, with confidence intervals crossing unity, suggesting that no single study had a disproportionate effect on the meta-analytic estimate. Heterogeneity was generally low to moderate and decreased when certain studies were excluded, reinforcing the strength of the overall conclusions.**

**Figure S3C: Use of AC mode**

**Figure S4: DOI plots**

Individual studies were excluded sequentially (Xirouchaki et al., 2008; Elganzay et al., 2014; Eixeira et al., 2015; Bosma et al., 2016; Botha et al., 2018; Bosma et al., 2025), but the aggregate risk ratio or statistical significance remained unchanged. In all leave-one-out analyses, confidence intervals crossed unity, indicating that no single study had a disproportionate impact on the total effect estimate. Heterogeneity remained low to moderate and was reduced when particular studies were excluded, bolstering the meta-analytic findings.

**Figure S4A Duration of ventilation:**

The Doi plot displays effect sizes (ES) plotted against standardized Z-scores for studies comparing proportional assist ventilation (PAV) with pressure support ventilation (PSV) during weaning from invasive mechanical ventilation. The plot shows noticeable asymmetry, with a predominance of studies on one side of the pooled effect estimate and a non-uniform distribution across Z-scores. The calculated Luis Furuya-Kanamori (LFK) index was **1.38**, indicating **minor asymmetry**. This suggests the possible presence of small-study effects or mild publication bias, although the degree of asymmetry is unlikely to substantially compromise the robustness of the meta-analytic findings for duration of ventilation.

**Figure S4B ICU duration:**

The Doi plot presents effect sizes (ES) plotted against standardized Z-scores for studies comparing proportional assist ventilation (PAV) with pressure support ventilation (PSV) during weaning from invasive mechanical ventilation, with ICU length of stay as the outcome. The distribution of studies appears largely symmetrical around the pooled effect estimate, with no evident directional clustering suggestive of publication bias. The calculated Luis Furuya-Kanamori (LFK) index was **−0.47**, indicating **no asymmetry**. This finding suggests a low likelihood of small-study effects or publication bias influencing the meta-analytic estimate for ICU duration.

**Figure S4C Mortality:**

The Doi plot illustrates effect sizes (ES) plotted against standardized Z-scores for studies comparing proportional assist ventilation (PAV) with pressure support ventilation (PSV) during weaning from invasive mechanical ventilation, with mortality as the outcome. The plot demonstrates clear asymmetry, with an uneven distribution of studies across the pooled effect estimate. The Luis Furuya-Kanamori (LFK) index was **−1.93**, indicating **major asymmetry**. This finding suggests the presence of substantial small-study effects or potential publication bias, which may influence the pooled mortality estimate and should be interpreted with caution.

**Figure S4D Patients requiring intubation:**

The Doi plot depicts effect sizes (ES) plotted against standardized Z-scores for studies comparing proportional assist ventilation (PAV) with pressure support ventilation (PSV) during weaning from invasive mechanical ventilation, with the proportion of patients requiring intubation as the outcome. The plot demonstrates pronounced asymmetry, with a marked imbalance in the distribution of studies across the pooled effect estimate. The Luis Furuya-Kanamori (LFK) index was −5.63, indicating major asymmetry. This substantial asymmetry suggests a high likelihood of small-study effects or publication bias, and the pooled estimate for this outcome should therefore be interpreted with considerable caution.

**Figure S4E: Use of Assist control device,**

The Doi plot presents effect sizes (ES) plotted against standardized Z-scores for studies comparing proportional assist ventilation (PAV) with pressure control ventilation (PCV) during weaning from invasive mechanical ventilation, with use of assist-control ventilation as the outcome. The plot demonstrates marked asymmetry, characterized by a skewed distribution of studies across the pooled effect estimate. The Luis Furuya-Kanamori (LFK) index was **−6.57**, indicating **major asymmetry**. This substantial asymmetry suggests a high probability of small-study effects or publication bias, and the pooled estimate for this outcome should therefore be interpreted with caution.

**Figure S5: Trial sequential analysis.**

**Figure S5A ICU Duration:**

Trial sequential analysis (TSA) of ICU duration is shown in **Figure S5A**. The required information size was calculated assuming an overall type I error of 5% and a type II error of 20%, using a two-sided graph and a model variance–based estimate of diversity. The control group mean and variance were derived from the included trials, as described in the *Methods* section. The cumulative sample size did not reach the required information size. Although the cumulative Z-curve crossed the conventional boundary for statistical significance in favor of proportional assist ventilation (PAV), it did not cross the trial sequential monitoring boundary for benefit or futility. When the accumulated data are less than the required information size, firm conclusions require crossing of the monitoring boundaries. Therefore, the TSA indicates that the evidence for a reduction in ICU duration remains **inconclusive**, and the observed effect may be susceptible to random error due to insufficient information size.

**Figure S5B Mortality:**

TSA of the secondary outcome, mortality, is shown in the figure. In this TSA, the required information size was calculated allowing for an overall type 1 error of 5%, a type 2 error of 20%, and assuming a control event proportion derived from the pooled control arms of the included trials, a prespecified relative risk reduction for mortality, and a model variance–based estimate of diversity as described in the Methods section. The accumulated sample size did not reach the required information size for mortality.

The TSA-adjusted confidence interval crossed unity, indicating statistical uncertainty around the true effect estimate. Because the accrued information was below the required information size, the cumulative z-curve was required to cross the trial sequential monitoring boundaries to confirm benefit, harm, or futility. The cumulative z-curve crossed only the conventional boundary but did not cross the trial sequential monitoring boundary for benefit, harm, or futility.

Overall, the TSA for mortality suggests that the current evidence remains inconclusive and underpowered, and additional adequately sized trials are required before a definitive conclusion regarding the effect of PAV on mortality can be drawn.

**Figure S5C Pt requiring intubation:**

TSA of the secondary outcome, patients requiring intubation, is shown in the figure. In this TSA, the required information size was calculated allowing for an overall type 1 error of 5% and a type 2 error of 20%, using a two-sided alpha-spending function. The control event proportion, anticipated relative risk reduction, and diversity-adjusted model variance were derived from the included trials, as detailed in the Methods section. The accrued sample size did not reach the required information size of 2638 patients.

Because the accumulated information was below the required information size, the cumulative z-curve was required to cross the trial sequential monitoring boundaries to establish benefit, harm, or futility. The cumulative z-curve crossed the conventional boundary in favor of PAV but did not cross the trial sequential monitoring boundary for benefit or futility.

Overall, the TSA for patients requiring intubation indicates that the available evidence is insufficient to draw firm conclusions. The findings suggest a potential signal favoring PAV; however, the results remain inconclusive due to sparse data and inadequate information size. Additional adequately powered randomized trials are required to confirm or refute the effect of PAV on the need for intubation.

**Figure S5D Use of AC mode:**

TSA of the secondary outcome, use of assist-control (AC) mode, is shown in the figure. In this TSA, the required information size was calculated allowing for an overall type 1 error of 5% and a type 2 error of 20%, using a two-sided alpha-spending function. The control event proportion, anticipated relative risk reduction, and diversity-adjusted model variance were derived from the included trials, as outlined in the Methods section. The accumulated sample size remained below the required information size for this outcome.

Because the accrued information was less than the required information size, the cumulative z-curve needed to cross the trial sequential monitoring boundaries to establish clear evidence of benefit, harm, or futility. The cumulative z-curve crossed the conventional boundary in favor of PAV but did not cross the trial sequential monitoring boundary for benefit or futility.

Overall, the TSA for use of AC mode suggests that the current evidence is suggestive but not conclusive. While the point estimate trends toward reduced reliance on AC with PAV, the available data remain insufficient to confirm a definitive effect, and additional adequately powered randomized trials are needed to reach a firm conclusion.

**Figure S5E Ventilation duration:**

TSA of the secondary outcome, ventilation duration is shown in this figure. In this TSA, the required information size was calculated allowing for an overall type 1 error of 5%, a type 2 error of 20%. The estimates for the control event proportion and diversity were taken from the included trials, as described in the “Methods” section. TSA of ventilation duration showed that the effect size exceeded the required information size of 280 patients. The cumulative z-curve crossed the conventional boundary and the trial sequential monitoring boundary for benefit in favor of PAV, suggesting conclusive, robust results. Therefore, TSA confirmed our meta-analysis providing convincing statistical evidence (supplementary file S5A-E).
